# Tofla virus: A newly identified *Nairovirus* of the Crimean-Congo hemorrhagic fever group isolated from ticks in Japan

**DOI:** 10.1038/srep20213

**Published:** 2016-02-11

**Authors:** Satoshi Shimada, Kotaro Aoki, Takeshi Nabeshima, Yu Fuxun, Yohei Kurosaki, Kazuya Shiogama, Takanori Onouchi, Miako Sakaguchi, Takeshi Fuchigami, Hokuto Ono, Kodai Nishi, Guillermo Posadas-Herrera, Leo Uchida, Yuki Takamatsu, Jiro Yasuda, Yutaka Tsutsumi, Hiromi Fujita, Kouichi Morita, Daisuke Hayasaka

**Affiliations:** 1 Department of Virology, 1-12-4 Sakamoto, Nagasaki, 852-8523, Japan; 2 Department of Emerging Infectious Diseases, 1-12-4 Sakamoto, Nagasaki, 852-8523, Japan; 3 Department of Pathology, Fujita Health University School of Medicine, 1-98 Dengakugakubo, Kutsukake-cho, Toyoake, Aichi, 470-1192, Japan; 4 Central Laboratory, Institute of Tropical Medicine, 1-12-4 Sakamoto, Nagasaki, 852-8523, Japan; 5 Department of Hygienic Chemistry, Graduate School of Biomedical Sciences, Nagasaki University, 1-14 Bunkyo-machi, Nagasaki, 852-8521, Japan; 6 Department of Radioisotope Medicine, Atomic Bomb Diseases Institute, Nagasaki University, 1-12-4 Sakamoto, Nagasaki, 852-8523, Japan; 7 Mahara Institute of Medical Acarology, 56-3 korekuni Aratano-cho, Anan, Tokushima 779–1510, Japan; 8 Leading graduate school program, Nagasaki University, 1-12-4 Sakamoto, Nagasaki, 852-8523, Japan

## Abstract

Ixodid ticks transmit several important viral pathogens. We isolated a new virus (Tofla virus: TFLV) from *Heamaphysalis flava* and *Heamaphysalis formsensis* in Japan. The full-genome sequences revealed that TFLV belonged to the genus *Nairovirus*, family *Bunyaviridae*. Phylogenetic analyses and neutralization tests suggested that TFLV is closely related to the Hazara virus and that it is classified into the Crimean-Congo hemorrhagic fever group. TFLV caused lethal infection in IFNAR KO mice. The TFLV-infected mice exhibited a gastrointestinal disorder, and positron emission tomography-computed tomography images showed a significant uptake of ^18^F-fluorodeoxyglucose in the intestinal tract. TFLV was able to infect and propagate in cultured cells of African green monkey-derived Vero E6 cells and human-derived SK-N-SH, T98-G and HEK-293 cells. Although TFLV infections in humans and animals are currently unknown, our findings may provide clues to understand the potential infectivity and to develop of pre-emptive countermeasures against this new tick-borne *Nairovirus*.

Ixodid (hard) ticks transmit several important viral pathogens, including those of the families *Bunyaviridae*, *Flaviviridae*, *Reoviridae*, *Rhabdoviridae* and *Orthomyxoviridae*^1^,^2^,^3^. Tick-borne *Bunyaviruses* include highly pathogenic viruses, such as the Crimean-Congo hemorrhagic fever virus (CCHFV), severe fever with thrombocytopenia syndrome virus (SFTSV) and Heartland virus^4^,^5^,^6^. However, neither efficient vaccines nor specific treatments against these tick-borne diseases are currently available. Instead, epidemiological surveys of ticks provide important information, such as the infection rates in ticks, identification of the tick species that harbor the viruses, distribution of the tick vectors and endemic area, which are used to guide pre-emptive countermeasures to prevent infections with these viruses through tick bites.

CCHFV is categorized as a biosafety level (BSL)-4 pathogen and causes severe hemorrhagic disease with up to 30% mortality in humans^4^. CCHFV is transmitted by *Hyalomma* ticks and is broadly distributed in western China, central Asia, the Middle East to southeastern Europe, and throughout Africa^4^. CCHFV belongs to the genus *Nairovirus* of the family *Bunyaviridae*^4^. The principal vector of *Nairovirus* is ticks, and there are seven serogroups in the genus *Nairovirus*: i) CCHF, ii) Nairobi Sheep Disease (NSD), iii) Dera Ghazi Khan, iv) Hughes, v) Qalyub, vi) Sakhalin and vii) Thiafora. The CCHF and NSD groups include pathogenic viruses in humans and domestic animals^4^,^7^.

SFTS is an emerging disease that was first reported in the rural areas of central China in 2009^5^ and has currently been identified in Korea and Japan^8^,^9^. The causative agent, SFTSV, belongs to the genus *Phlebovirus* of the family *Bunyaviridae*. SFTSV has been detected in ticks in China, including *Haemaphysalis longicornis*^10^. An SFTS case was identified in Japan in 2013, and more than 140 cases of SFTS have been identified in western Japan. However, epidemiological information on SFTSV in ticks has not been fully elucidated in Japan. Therefore, we attempted to survey the tick species, infection rates and endemic areas of SFTSV in ticks distributed in Japan.

An epidemiological survey identified a new infectious agent that exhibited a fatal infection in IFNAR KO mice, which is a useful animal for the evaluation of tick-borne *Bunyaviruses*, including SFTSV, CCHFV and Hazara virus (HAZV)^11^,^12^,^13^. This study demonstrated the experimental findings of the isolation, classification, and *in vivo* and *in vitro* infectivity of this newly identified infectious agent, Tofla virus (TFLV).

## Results

### Virus isolation from *Haemaphysalis* ticks in Japan

**Figure 1:**
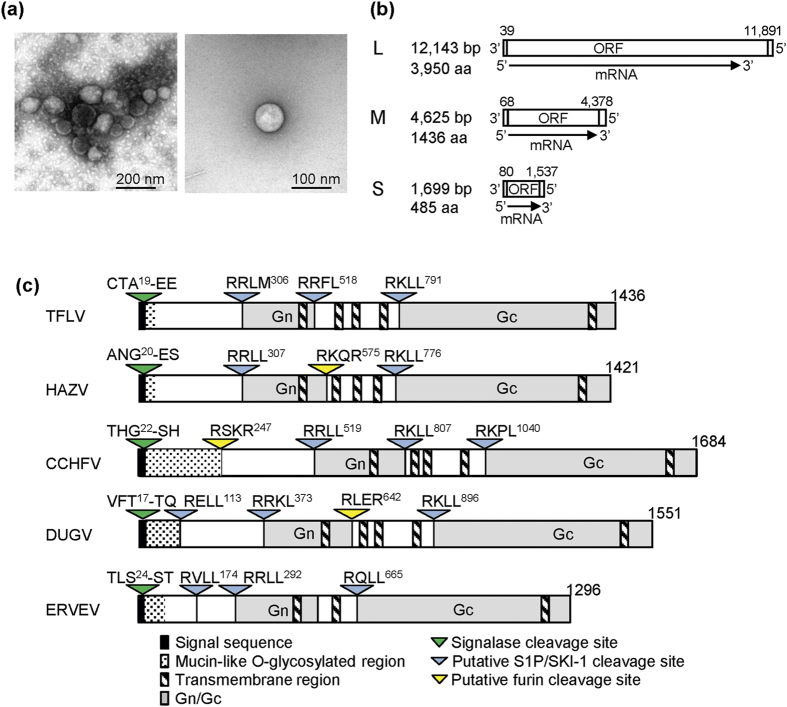
(**a**) Electron micrograph of TFLV. (**b**) Schematic diagram of three segments (L, M and S) of TFLV. (**c**) Schematic diagram of cleavage sites in the glycoproteins of the genus *Nairovirus*. HAZV, Hazara virus; CCHFV, Crimean–Congo hemorrhagic fever virus; DUGV, Dugbe virus; ERVEV, Erve virus.

Inoculations with homogenized tick pools of *H. flava* collected in Tokushima in 2013 and *H. formosensis* collected in Nagasaki in 2014 (Supplementary Figure 1) resulted in fatal outcomes in 129 strain background IFNAR KO (A129) mice at 3 days post-inoculation (pi). Homogenized spleen samples collected from the dead mice were filtrated and re-inoculated in A129 mice, and these re-inoculated mice died. Virus-like particles with diameters of approximately 100 nm were detected in the culture fluids (CFs) of Vero E6 cells after inoculation with a spleen sample of a mouse inoculated with a tick homogenate (*H. flava* in Tokushima) (Fig. 1a). These observations suggested that filterable infectious agents, i.e., TFLV, were included in the homogenized tick samples. Two strains of TFLV from *H. flava* in Tokushima and *H. formosensis* in Nagasaki were named Tok-Hfla-2013 and Nag-Hfor-2014, respectively.

### TFLV belongs to the genus *Nairovirus* of the family *Bunyaviridae*

We next determined the genome sequence of the TFLV using next-generation sequencing (NGS) and general sequencing. The genome had three segments with homologous sequences of the family *Bunyaviridae* (Fig. 1b). Two strains of Tok-Hfla-2013 and Nag-Hfor-2014 showed the same genome sizes for the L (12,143 bp), M (4,625 bp) and S (1,699 bp) segments. The deduced proteolytic sites in the glycoproteins Gn and Gc (i.e., the M segment) of TFLV were similar to the conserved sites in *Nairoviruses*, such as HAZV, CCHFV, Dugbe virus (DUGV) and Erve virus (ERVEV) (Fig. 1c). These results suggested that TFLV belongs to the genus *Nairovirus*.

### TFLV belongs to the CCHF group

**Figure 2:**
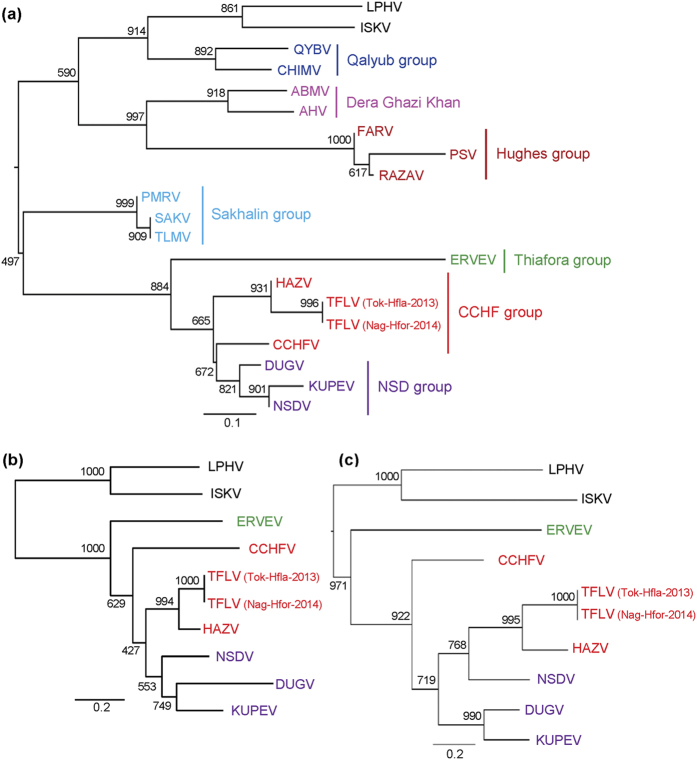
Phylogenetic trees of the genus *Nairovirus*. The phylogenetic trees were constructed based on the conserved amino acid sequences (**a**) Phylogenetic tree based on the L segment (amino acids 2218–2535 in the L segment of TFLV) using the substitution model LG + I + G. (**b**) Phylogenetic tree based on the M segment (amino acids 791–948 in TFLV) using the substitution model LG + G. (**c**) Phylogenetic tree based on the sequences of the S segment (2–179 of TFLV) using the substitution model LG + G. *Nairoviruses* are designated by the following abbreviations: ABHV, Abu Hammad virus; AMV, Abu Mina virus; CCHFV, Crimean–Congo hemorrhagic fever virus; CHIMV, Chim virus; DUGV, Dugbe virus; ERVEV, Erve virus; HAZV, Hazara virus; ISKV, Issyk-Kul virus; KUPEV, Kupe virus; LPHV, Leopards Hill virus; NSDV, Nairobi sheep disease virus; PMRV, Paramushir virus; PSV, Punte Salinas virus; QYBV, Qalyub virus; RAZAV, Raza virus; SAKV, Sakhalin virus; and TLMV, Tillamok virus.

The identities of the nucleotide sequences between the two strains of TFLV were 99.0, 97.2 and 99.1% in the S, M and L segments, respectively. The identities of the amino acid sequences were 100, 99.0 and 99.6% in the S, M and L segments, respectively. The phylogenetic analyses revealed that the two strains of TFLV clustered with HAZV (Fig. 2 and Supplementary Figure 2). The identities of the amino acids between two strains of TFLV and HAZV were 75.7%, 61.2–61.3% and 21.1–21.7% in the S, M and L segments, respectively.

The focus-reduction neutralization test (FRNT) using TFLV (Tok-Hfla-2013) demonstrated that anti-TFLV and anti-HAZV sera had 1:640 and 1:20 neutralization titers against TFLV, respectively (Table 1). The plaque reduction neutralization test (PRNT) using HAZV showed that anti-TFLV and anti-HAZV sera had 1:160 and 1:80 neutralization titers against HAZV (Table 1). These results suggest that the anti-TFLV and anti-HAZV sera exhibited cross-neutralization between TFLV and HAZV infections.

Of note, HAZV belongs to the CCHF serogroup^13^,^14^. Therefore, these data suggest that TFLV belongs to the CCHFV serogroup with HAZV.

### Pathogenicity of TFLV infection in A129 mice

**Figure 3:**
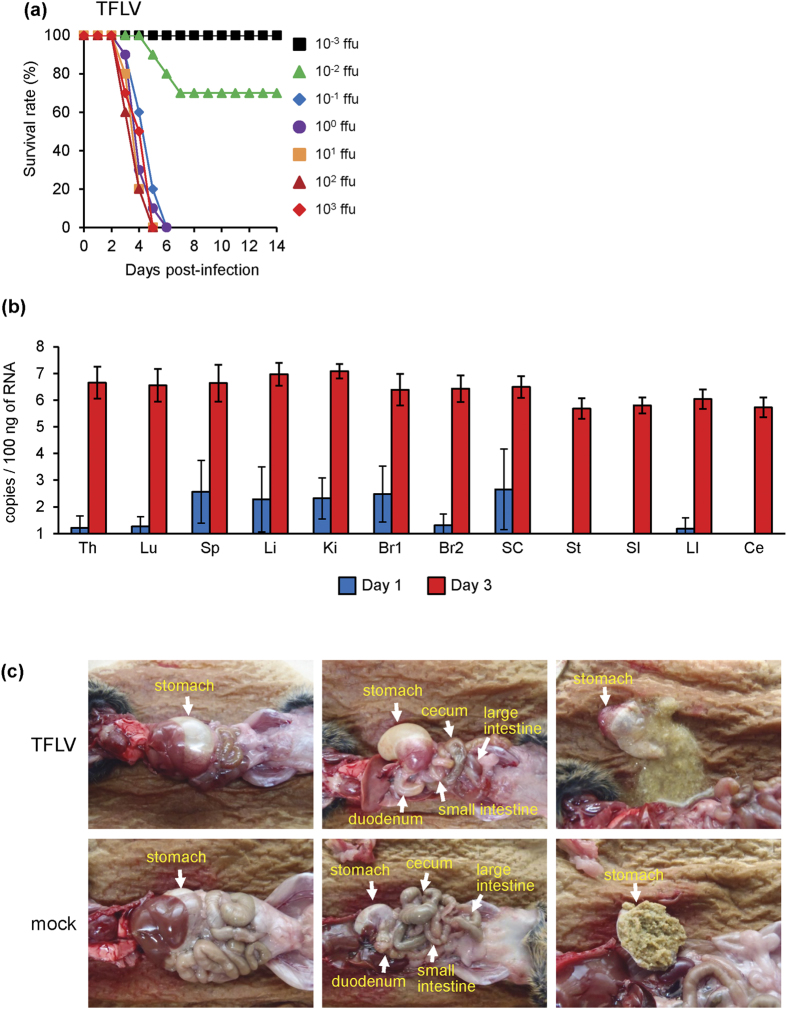
(**a**) Survival curves of A129 mice (n = 10) subcutaneously inoculated with 10^−3^ and 10^3^ ffu of TFLV (Tok-Hfla-2013). The mice were observed for 14 days, and no mice died after 14 days. (**b**) Viral loads in the tissues of A129 mice infected with 10^2^ ffu of TFLV (Tok-Hfla-2013). The copy numbers in the thymus (Th), lung (Lu), spleen (Sp), liver (Li), kidney (Ki), brain cortex (Br1) and non-cortex (Br2), spinal cord (SC), stomach (St), small intestine (SI), large intestine (LI) and cecum (Ce) of mice 1- and 3-days post-infection were determined using real-time RT-PCR (n = 5). (**c**) Gross pathology and viral loads of A129 mice inoculated with TFLV (Tok-Hfla-2013). Internal organs of TFLV-and mock-infected A129 3 days pi.

TFLV was identified from dead A129 mice inoculated with the tick samples. Therefore, we next examined the pathogenicity of the TFLV infection in A129 mice. Following subcutaneous inoculation with TFLV (Tok-Hfla-2013), the mice began to die at 3 days pi, and all mice died by day 6 following infection with titers containing more than 10^−1^ focus-forming units (ffu) infections (Fig. 3a). The LD_50_ of TFLV was 10^−1.7^ ffu (Table 2). The mean survival times of the TFLV infections with the 10^−1^ to 10^3^ ffu that showed 100% mortality were 3.8 ± 0.56 to 4.7 ± 0.68 days (Table 2). The mice that died exhibited acute clinical signs, such as slow movement, ataxia, piloerection, weight loss, and loose stool at 2 days pi, whereas the mice that survived did not exhibit any clinical signs.

Viral RNAs were detected in some tissues at 1-day pi and increased to as many as 10^6^ to 10^7^ copies/100 ng of RNA in every tissue (Fig. 3b) at 3 days pi, indicating that TFLV rapidly replicated in these tissues of the A129 mice. There were no significant differences in the viral RNA levels between tissues at 3 days pi (Fig. 3b).

Gross pathology revealed gastric and intestinal distensions, reduced stools in the intestine and reduced cecum size in the TFLV-infected mice (Fig. 3c). The stomach content of TFLV-infected mice was in liquid form, whereas that of mock-infected mice was in solid form (Fig. 3c).

**Figure 4:**
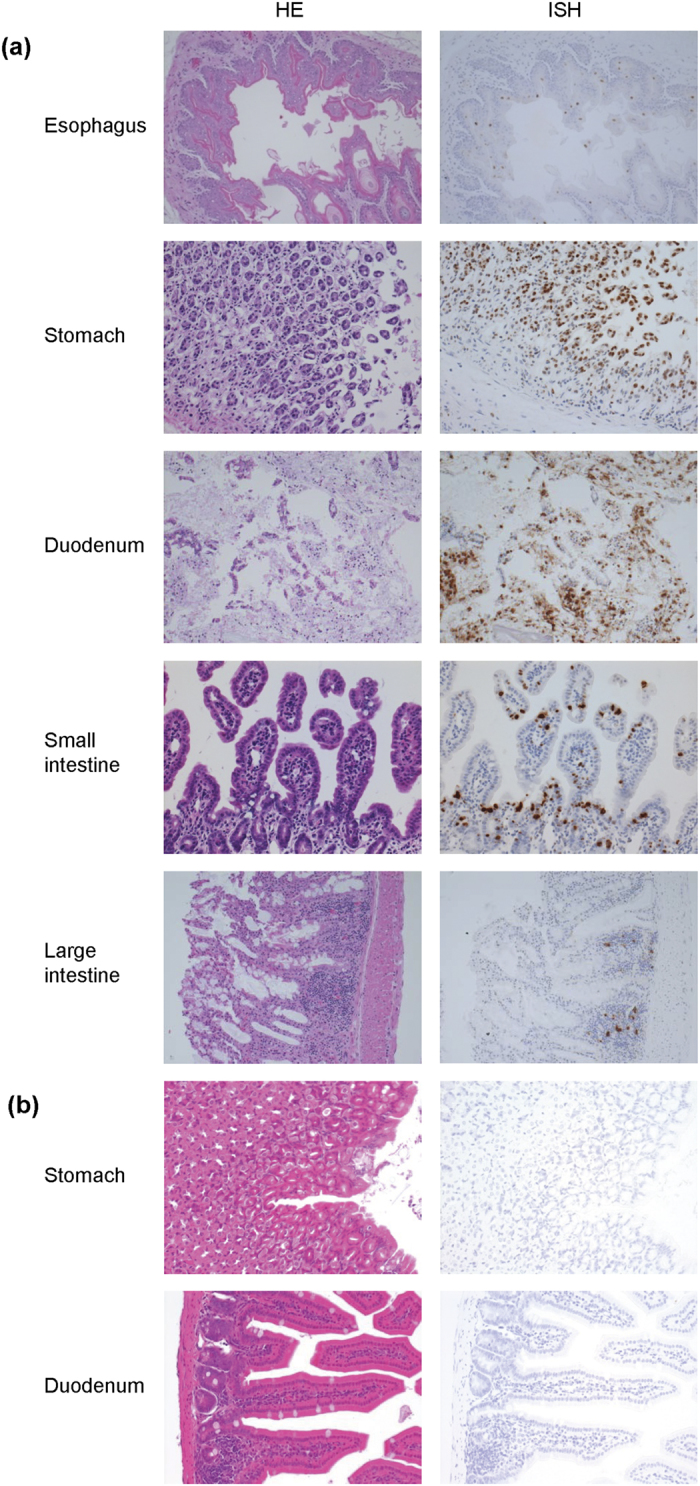
Histological and ISH features of the gastrointestinal tract. TFLV (Tok-Hfla-2013)-infected (**a**) and mock-infected (**b**) A129 mice were dissected at 3 days pi. Left panels: hematoxylin and eosin. Right panels: ISH using AT-tailed sense and antisense cocktail probes (positive signals are labeled in brown). The gastric and duodenal mucosae highly involved by the viral infection show marked erosive change. Non-eroded small intestinal mucosa reveal positive signals in both the crypts and villi. Some keratinocytes in the esophageal mucosa are also involved. In the large bowel mucosa, the germinal center of the lymphoid follicles represents the site of infection. The gastroduodenal mucosa in mock-infected control animals demonstrates normal histology without viral infection.

The histopathological examination revealed that the viral genomic RNA was prominently detected in epithelial cells, in particular, in the stomach, duodenum and small intestine (Fig. 4a). Erosive change of the gastric and duodenal mucosae was evident, and the viral genome was densely accumulated in these eroded areas. The viral genome was also detected in non-eroded small bowel mucosa and the germinal center of the colonic lymphoid follicles. Esophageal squamous mucosa was also mildly virus-infected. No lesions or signals were demonstrated in the mock-infected mice (Fig. 4b).

**Figure 5:**
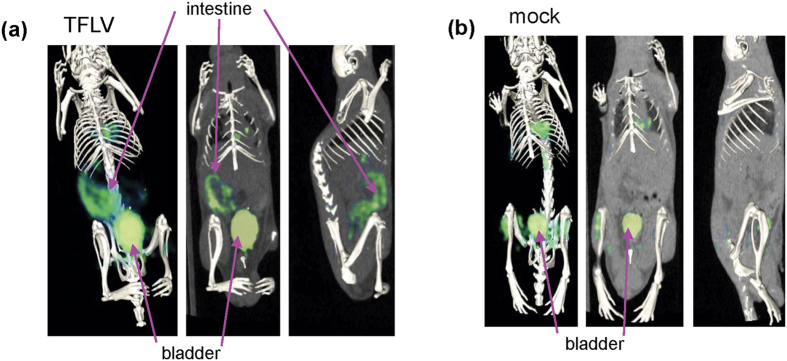
Representative whole body PET/CT images. TFLV (Tok-Hfla-2013)-infected (**a**) and mock-infected (**b**) A129 mice were observed at 3 days pi (n = 4). The PET/CT images were acquired 30–60 min after intravenous injection of ^18^F-FDG (10 MBq in 400 μl of saline per mouse).

The ^18^F-fluorodeoxyglucose (FDG) positron emission tomography-computed tomography (PET/CT) images showed ^18^F-FDG uptake in the intestinal tract (Fig. 5a). No significant uptake of ^18^F-FDG in the intestinal tract was observed in the mock-infected mice (Fig. 5b). These results suggest that the TFLV infection caused gastrointestinal disorders in A129 mice.

### Infectivity of TFLV in human cell lines

**Figure 6:**
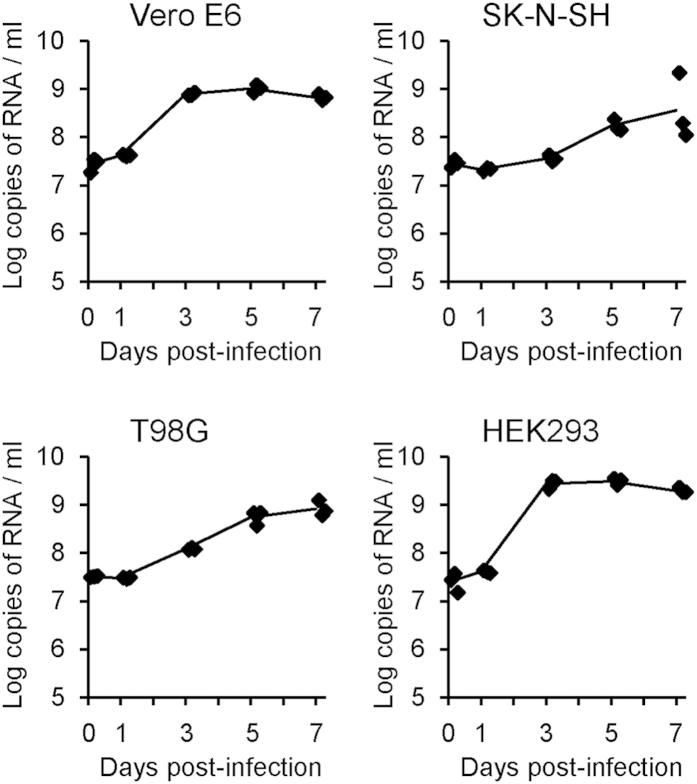
TFLV (Tok-Hfla-2013) propagation in the Vero E6, SK-N-SH, T98-G and HEK-293 cells. CFs were harvested at 0, 1, 3, 5 and 7 days pi. Growth curves in the CFs are indicated by the viral RNA copy numbers.

We next examined whether TFLV could infect and propagate in human cells. African green monkey-derived Vero E6 and human-derived SK-N-SH, T98-G and HEK-293 cells were inoculated with TFLV (Tok-Hfla-2013), and viral RNAs in the CFs were detected. The viral RNAs in the CFs significantly increased at 1 to 3 days pi in the Vero E6 cells (Fig. 6). Increased viral RNAs were observed in the SK-N-SH, T98-G and HEK-293 cells at 3 to 5 days pi (Fig. 6). These results suggest that TFLV can infect and propagate in these cells, although whether TFLV indeed infects humans is not certain from these results.

## Discussion

This study identified a new TFLV from ticks in Japan. The TFLV was closely related to HAZV, which belongs to the CCHF group of the genus *Nairovirus*. Therefore, the TFLV is suggested to be a new virus in the CCHF group. HAZV was isolated from *Ixodes redikorzevi* collected in Pakistan in 1964^15^, and no further virus strains have been reported since that time. HAZV infectivity and pathogenicity have not been reported in humans. Thus, HAZV can be used as a BSL-2 pathogen, whereas CCHFV must be used in a BSL-4 facility. We used TFLV in a BSL-3 facility because its pathogenic potential in humans is not clear. Further investigation of human TFLV pathogenicity is required.

We isolated two strains, Tok-Hfla-2013 and Nag-Hfor-2014, from different tick species in different areas. The distance between Tokushima and Nagasaki is approximately 500 km. The tick species *H. flava* and *H. formosensis* feed on wild animals, such as deer and wild boars, and they also feed on humans. *H. flava* is distributed over a large area of Japan, whereas *H. formosensis* has been collected in the western and southern regions of Japan, but not in the eastern and northern areas^16^,^17^. Therefore, TFLV may be distributed throughout Japan and may naturally circulate within ticks and animals.

TFLV exhibited lethal infection in A129 mice. We also attempted to infect immunocompetent mice with TFLV, such as B6 mice, but these mice did not exhibit any clinical signs (data not shown). Thus, IFN-I responses are likely to be important for protection against TFLV in mice. Previous reports have shown that IFNAR KO mice are useful animal models for *in vivo* infections with CCHFV and HAZV in the CCHF group^12^,^13^, and it has been suggested that inoculating mice with HAZV may act as a surrogate model for testing antiviral agents against CCHFV^13^,^14^. Thus, the TFLV infection of mice may also provide a potential model for such testing against CCHFV.

The cause of death in the TFLV-infected A129 mice was uncertain, but a severe gastrointestinal disorder, including indigestion, was observed in the infected mice. Apparently ^18^F-FDG was taken up in the intestinal tract, but not in the stomach, although pathological lesions and viral infections were similarly observed in both tissues. ^18^F-FDG is a glucose analog, and its uptake is a marker for tissue glucose uptake, which closely correlates with certain types of tissue metabolism^18^. Thus, glucose metabolism in the intestine may be a key factor of the severe gastrointestinal disorder correlated with the lethal infection in the TFLV-infected mice. Further elucidations of the mechanism of ^18^F-FDG accumulation in the intestine may provide a novel approach to understanding the disease due to the TFLV-infection in this mouse model.

SFTSV is recently identified emerging virus. The earliest case of all of the reported SFTS cases worldwide was from a patient in Nagasaki, Japan, who was infected in 2005 9. SFTS cases are sporadically distributed in Japan. Therefore, SFTSV has likely existed in this country for a long time, but the disease was not identified because of the rarity of cases. This observation suggests that TFLV might have also caused a disease outbreak that was not identified. Our study demonstrated that TFLV infected and replicated in human cells, but it is unclear if actual viral infections occur in humans. Further investigations, including seroepidemiological surveys using human and animal samples, are an important priority to determine whether TFLV causes infectious diseases in humans and animals. In addition, *in vitro* experiments using more cell lines, including other tissue-derived human cell lines, such as gastrointestinal cells, and other animal-derived cell lines, will provide useful information for understanding the infectivity of TFLV in mammals.

## Methods

### Virus and cells

The HAZV and the YG-1 strain of SFTSV were kindly provided by Roger Hewson, Public Health England, and Ken Maeda, Yamaguchi University, respectively. Stock viruses of TFLV and SFTSV were prepared from a cell culture medium of Vero E6 (African green monkey kidney) cells. The infectious titers of the TFLV and SFTSV stocks were determined using a focus-forming assay. The HAZV stock virus was prepared in SW13 (Human adenocarcinoma) cells. The infectious titer of the HAZV stock was determined using a plaque-forming assay. Vero E6, SW13, SK-N-SH (human neuroblastoma), T98-G (human glioblastoma multiform) and HEK-293 (human embryonic kidney) cells were maintained in Eagle’s Minimal Essential Medium (EMEM; Nissui Pharmaceutical Co., Tokyo, Japan) containing 10% fetal bovine serum (FBS). All experiments using live TFLV and SFTSV were performed in a BSL-3 laboratory at Nagasaki University according to standard BSL-3 guidelines. The experiments using live HAZV were performed in a BSL-2 laboratory.

### Focus-forming assay

Confluent Vero E6 cells were inoculated with serially diluted culture supernatants of TFLV and SFTSV stocks and incubated in 2% FCS EMEM containing 1% methyl cellulose 4,000 (Wako Pure Chemical Industries, Ltd., Tokyo, Japan) for 5 days. The viral foci of TFLV were detected using TFLV antiserum from the TFLV-infected B6 mice, peroxidase-conjugated anti-mouse IgG (American Qualex, CA, USA) and DAB substrate (Wako Pure Chemical Industries, Ltd., Tokyo, Japan). The viral foci of SFTSV were detected using an SFTSV antiserum from the recovered SFTS cases, peroxidase-conjugated anti-human IgG (American Qualex, CA, USA) and DAB substrate. Viral titers were expressed as ffu/ml. The experiment using human serum was performed with the approval of the ethics committee of the Institute of Tropical Medicine, Nagasaki University (approval number: 140829129).

### Plaque-forming assay

Confluent SW13 cells were inoculated with serially diluted culture supernatants of the HAZV stock and incubated in 0.7% FBS MEM containing 0.7% agarose for 4 days. The cells were fixed with a methanol/acetic acid mixture (5:1) and stained with 0.3% amido black. The viral titers were expressed as pfu/ml.

### Mice

A129 mice were purchased from B&K Universal Limited and were mated in the facility at Nagasaki University. The adult (older than eight weeks old) A129 mice were subcutaneously inoculated with 10^−3^ to 10^3^ ffu of TFLV (Tok-Hfla-2013 strain) (n = 10) in EMEM containing 2% FBS. The mice were weighed daily and observed for clinical signs of disease. The B6 mice were purchased from CLEA Japan, Inc. The animal experiments were performed in accordance with the recommendations in the Fundamental Guidelines for the Proper Conduct of Animal Experiments and Related Activities in Academic Research Institutions under the jurisdiction of the Ministry of Education, Culture, Sports, Science and Technology. The Animal Care and Use Committee of the Nagasaki University approved all of the experimental protocols (approval number: 1401201115, 1408051166).

### TFLV isolation from ticks

*H. flava* and *H. formosensis* were collected by flagging in Tokushima in 2013 and in Nagasaki in 2014, respectively. The pooled ticks of *H. flava* (9 nymphs) and *H. formosensis* (30 nymphs) were homogenized using Micro Smash^TM^ MS-100 R (TOMY DIGITAL BIOLOGY CO., LTD, Tokyo, Japan) with one stainless bead (4.8 Ø) and 0.5 ml of 2% FBS EMEM per reaction tube at 4,500 rpm for 15 seconds at 4 °C. A total of 100 μl of the samples was used to intraperitoneally inoculate adult A129 mice. The spleens were collected from the dead mice after 3 days and homogenized in 2% FBS EMEM using the same procedure as the tick homogenization. The supernatants of the homogenized spleen samples were inoculated in Vero E6 cells, and the CFs were harvested after 6 days.

### Electron microscopy

The CFs from the TFLV-infected Vero E6 cells were mixed with PEG 6000 (Wako) and NaCl (Wako, Tokyo, Japan) and stirred at 4 °C for 2 hours. The supernatant was centrifuged at 12,000 G for 1 hour. The PEG and virus pellet was dissolved in 4 ml of STE buffer and centrifuged at 12,000 G for 10 minutes. The supernatant sample was loaded on a sucrose gradient (50% to 15%) and centrifuged at 100,000 G at 4 °C overnight. The turbid band containing virions was collected. The sample was dissolved in STE buffer and centrifuged at 200,000 G for 4 hours. The pellet at the bottom of the tube was dissolved in STE buffer and centrifuged again to completely remove the sucrose. The pellet was dissolved in phosphate-buffered saline (PBS), loaded on a 200-mesh copper grid with carbon-coated plastic film (Nisshin EM, Tokyo, Japan), and negatively stained with 10 μl of a uranyl acetate solution (1.5%, w/v) for 10 s. The morphology of the sample was observed on a JEM-1230 (JEOL Ltd., Tokyo, Japan), with a 80 kV acceleration voltage, and imaged using a 2k × 2k Veleta CCD camera (Olympus Soft Imaging Solutions, Lakewood, CO, USA).

### TFLV genome sequence

RNA was extracted from the homogenized mouse spleens using a RNeasy Lipid Tissue Mini Kit (Qiagen, Hilden, Germany). Next-generation sequencing (NGS) was performed using a GS Junior 454 (Roche Diagnostic Cooperation, Branford, CT, USA). The viral genome sequences were assembled and analyzed using CLC Genomics Workbench software. The homolog sequences with the *Nairovirus* were determined using BLAST (http://blast.ncbi.nlm.nih.gov). The terminal regions of the genome were determined using the Sanger method using primers that were designed from the terminal region of the Nairovirus sequence, and the 5′-Rapid Amplification of cDNA End (5′-RACE) method was performed using a 5′-Full RACE Core Set (Takara Bio, Inc., Tokyo, Japan). The primers were designated using PRIMER3-web (http://primer3.ut.ee). PCR amplification was performed using Tks Gflex™ DNA Polymerase (Takara Bio, Inc., Tokyo, Japan), and Sanger sequencing was performed using a 3130 × l Genetic Analyzer or 3100 Avanti Genetic Analyzer (Applied Biosciences, CA, USA). The GenBank accession numbers of Tok-Hfla-2013 are LC008510 (S segment), LC0008511 (M segment), and LC0008512 (L segment), and the numbers of Nag-Hfor-2014 are LC030113 (S segment), LC030114 (M segment) and LC030115 (L segment).

### Phylogenetic analysis

The genome sequences were assembled and analyzed using Phred/Phrap/Consed software. The viral open reading frames and translated amino acid sequences were analyzed using EMBOSS software ver. 6.3.1. The amino acid sequences were aligned using dialign-tx 1.0.2 software^19^ and processed using TrimAl 1.2 software^20^. The amino acid sequences based on full-genome sequences were aligned using mafft v7.245^21^. The phylogenetic trees were constructed based on the conserved region of the amino acid sequences (Fig. 2) and based on the full amino acid sequences (Supplementary Figure 2). The substitution models were determined by Protest v3.2^22^ and Protest v3.4.1. The genetic distance among *Nairoviruses* was deduced using the maximum likelihood model by PhyML v3.0.1^23^. The phylogenetic trees were processed using FigTree 1.4.2 tree viewer software. The following accession codes were obtained for phylogenetic and homology analyses: Abu Hammad virus (AAQ93043.1), Abu Mina virus (AAQ93044.1), CCHFV (NP_950237.1, YP_325663.1, NP_950235.1), Chim virus (KF801656.1), DUGV (NP_690576.1, NP_690575.1,NP_690574.1), ERVEV (AFH89034.1, AFH89033.1, AFH89032.1), Farallon virus (AAQ93048.1), HAZV (KC344857.1, DQ813514.1, DQ076419.1), Issyk-Kul virus (AII79373.1), Leopards Hill virus (YP_009111284.1), NSD virus (ACH99797.1), Paramushir virus (AHH24352.1), Punte Salinas virus (AAQ93052.1), Qalyub virus (AAQ93053.1), Raza virus (AAQ93054.1), Sakhalin virus (AHH24354.1) and Tillamok virus (AAQ93055.1).

### Neutralization test

The B6 mice were subcutaneously infected with 10^4^ ffu of the TFLV (Tok-Hfla-2013), 10^3^ pfu of the HAZV or 10^6^ ffu of the SFTSV, and blood samples were collected after 3 weeks pi. The sera were collected from the blood samples. The neutralizing antibody titers against TFLV and SFTSV were determined using 50% FRNT, and those against HAZV were determined using 50% PRNT. The serially diluted sera were mixed with TFLV, HAZV or SFTSV and incubated at 37 °C for 1 hour. The Vero E6 cells were inoculated with mixtures of TFLV or SFTSV and incubated for 5 days. The SW13 cells were inoculated with a mixture of HAZV and incubated for 4 days. The focus and plaque staining were performed as described above. The neutralizing titers were determined as the reciprocal of the highest serum dilution that reduced the viral foci of TFLV and SFTSV or the plaque counts of HAZV by 50%.

### Copy numbers of TFLV RNA in mouse tissues

The viral replications in the TFLV-infected mice were detected by the viral copy numbers as shown in previous studies for *Bunyavirus* infections^11^,^13^,^24^,^25^,^26^. The mice were subcutaneously infected with 10^2^ ffu of TFLV (Tok-Hfla-2013), and five mice per group were sacrificed at 1 and 3 days pi. The thymus, lungs, spleen, liver, kidneys, small intestine, colon, cecum, brain and spinal cord were removed following perfusion with cold PBS. The small intestine, colon and cecum were washed with cold PBS to remove the stool. The brains were divided into two fractions, the brain cortex and non-cortex tissues. These tissues were immediately submerged in RNAlater (Ambion, CA, USA) and stored at −80 °C until they were used. The total RNA was extracted using an RNeasy Lipid Tissue Mini Kit (Qiagen, Hilden, Germany). The viral copy numbers were examined by using real-time RT-PCR. The TFLV-specific primers and probes were designated based on the M segment using PRIMER3-web. The forward primer was HSKV_M1F: 5′-GCATACACCATGTCCGTGAG-3′, the reverse primer was HSKV_M1R: 5′-GGAAAGTTCCTGAGCTGCAC-3′ and the PrimeTime® qPCR probe was HSKV_M1IN: 5′-/56-FAM/CACCACCAC/ZEN/CTGCATAACAG/3IABkFQ/-3′ (Integrated DNA Technologies, IA, USA). The real-time RT-PCR reactions were performed using a One Step PrimeScript RT-PCR Kit (Takara Bio, Inc., Tokyo, Japan) in a 7500 Real-time PCR System (Applied Biosystems, CA, USA). The copy numbers were calculated as a ratio of the copy numbers of a standard control.

### Histopathological study and *In situ* hybridization (ISH)

The mice were subcutaneously infected with 10^2^ ffu of TFLV (Tok-Hfla-2013), and five mice per group of the A129 mice were anesthetized and perfused with 10% phosphate-buffered formalin. The fixed tissues were routinely embedded in paraffin, sectioned, and stained with hematoxylin and eosin. The viral RNA was detected using ISH in formalin-fixed, paraffin-embedded (FFPE) sections using the AT-tailing method for high-sensitivity detection^27^. The FFPE sections mounted on aminosilane-coated glass slides (Matsunami, Tokyo, Japan) were heated in a pressure pan cooker in 10 mM citrate buffer, pH 6.0 for 10 min, followed by digestion with 0.1 μg/mL proteinase K for 15 min at 37 °C. The endogenous biotin activity was quenched using a biotin blocking system (Dako, Carpinteria, CA, USA), and the sections were hybridized overnight at 50 °C with 0.01 pmol/mL AT-tailed oligonucleotide cocktail sense or antisense probes against the L segment (sense: 5′-GTCTGAGTGGCCAGACTCAAGAGTGGTCACAC TAGTAGTGATATATATATATATATATAT-3′, antisense: 5′-CACTACTAGTGTGACCA CTCTTGAGTCTGGCCACTCAGACATATATATATATATATATAT-3′), M segment (sense: 5′-TGGCTTCACTTCACTACCCTCTGTTATGCAGGTGGTGGTGATATATAT ATATATATATAT-3′, antisense: 5′-CACCACCACCTGCATAACAGAGGGTAGTGAA GTGAAGCCAATATATATATATATATATAT-3′) and S segment (sense: 5′-GGAGTTCC ACGCCTGGTTCAAGGCCTATTCAGATAAGCACATATATATATATATATATAT-3′, antisense: 5′-GTGCTTATCTGAATAGGCCTTGAACCAGGCGTGGAACTCCATATA TATATATATATATAT-3′) segments of the viral RNA. The sections were rinsed in 1× saline sodium citrate (SSC) after hybridization followed by 0.1× SSC for 10 min at 55 °C. “Gene Frame” (Thermo Fisher Scientific, Yokohama, Japan) was attached to each slide for exposure to the AT-tailing mixture containing nucleotides, biotin-16-dUTP and Gene Taq DNA polymerase for 10 min at 60 °C. The Gen Point System (Dako, Carpinteria, CA, USA) was employed for signal amplification as follows. One thousand-fold diluted streptavidin-horseradish peroxidase (SA-HRP) was applied to the sections for 15 min at room temperature (RT). The biotinylated tyramides were reacted for 15 min at RT. SA-HRP was challenged again for 15 min at RT. The reaction products were visualized in 20 mg/dL diaminobenzidine tetrahydrochloride and 0.006% hydrogen peroxide in 50 mM Tris-HCl buffer, pH 7.6, and the nuclei were lightly counterstained with hematoxylin.

### PET/CT imaging

PET/CT images were acquired using Triumph combined PET/SPECT/CT systems (TriFoil Imaging, Inc., CA, USA). The A129 mice were subcutaneously infected with 10^2^ ffu of TFLV (Tok-Hfla-2013) and administered 10.2–11.4 of MBq ^18^F-FDG (Nihon Medi-Physics Co., Ltd. Kurume, Japan) intravenously in the tail vein 3 days pi. The mice were anesthetized with 1.5% isoflurane, and CT acquisitions were performed for anatomical reference. Subsequently, PET acquisitions were performed for 30 min starting 30 min after ^18^F-FDG injection. The PET data were reconstructed using a 3D-Maximum-Likelihood Expectation Maximization (MLEM) algorithm (60 iterations). The acquired PET and CT data were processed using VIVID™ (TriFoil Imaging, Inc.).

### Growth curves of TFLV in cultured cells

A total of 10^5^ cells per well of the Vero E6, SK-N-SH, T98-G and HEK-293 cells were inoculated with 10^3^ ffu of TFLV (Tok-Hfla-2013) at a multiplicity of infection of 0.01 in 12-well plates. The experiment was performed in triplicate. The CFs and cells were harvested at 0, 1, 3, 5 and 7 days pi. RNAs were extracted from the CFs using a QIAamp Viral RNA Mini Kit (Qiagen, Hilden, Germany). The viral copy numbers were examined using real-time RT-PCR as described above.

## Additional Information

**How to cite this article**: Shimada, S. *et al.* Tofla virus: A newly identified *Nairovirus* of the Crimean-Congo hemorrhagic fever group isolated from ticks in Japan. *Sci. Rep.* **6**, 20213; doi: 10.1038/srep20213 (2016).

## Supplementary Material

Supplementary Figures

## Figures and Tables

**Table 1:**
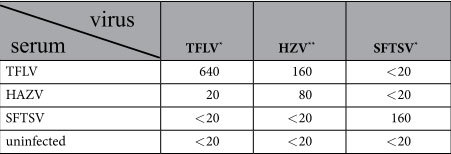
Neutralizing titers of mice sera infected with TFLV and SFTSV.

^*^50% FRNT.

^**^50% PRNT.

**Table 2:** Survival days of dead mice infected with TFV, HZV and SFTSV.

	LD_50_	Infection dose*	Fatality**	MST*** ± 95% CI
TFLV	10^−1.7^	10^−2^	3/10	6.0 ± 2.40
		10^−1^	10/10	4.7 ± 0.68
		10^0^	10/10	4.3 ± 0.59
		10^1^	10/10	4.0 ± 0.48
		10^2^	10/10	3.8 ± 0.56
		10^3^	10/10	4.2 ± 0.66

^*^ffu.

^**^Fatal number/total number.

^***^Mean of survival time: days.
