# Supplementary material for: Tofla virus: A newly identified Nairovirus of the Crimean-Congo hemorrhagic fever group isolated from ticks in Japan
Source: Sci Rep. 2016 Feb 11;6:20213. doi: 10.1038/srep20213 (PMC4809068; doi:10.1038/srep20213)

Supplementary information for

Title

Tofla virus: A newly identified *Nairovirus* of the Crimean-Congo hemorrhagic fever group isolated from ticks in Japan

Authors

Satoshi Shimada[1,8], Kotaro Aoki[1], Takeshi Nabeshima[1], Yu Fuxun[1], Yohei Kurosaki[2], Kazuya Shiogama[3], Takanori Onouchi[3], Miako Sakaguchi[4], Takeshi Fuchigami[5], Hokuto Ono[5], Kodai Nishi[6], Guillermo Posadas-Herrera[1+], Leo Uchida[1], Yuki Takamatsu[1], Jiro Yasuda[2,8], Yutaka Tsutsumi[3], Hiromi Fujita[7], Kouichi Morita[1,8] and Daisuke Hayasaka[1,8]*

Affiliation

[1]Department of Virology, [2]Department of Emerging Infectious Diseases, [4]Central Laboratory, Institute of Tropical Medicine, [8]Leading graduate school program, Nagasaki University, 1-12-4 Sakamoto, Nagasaki, 852-8523, Japan.

[3]Department of Pathology, Fujita Health University School of Medicine, 1-98 Dengakugakubo, Kutsukake-cho, Toyoake, Aichi, 470-1192, Japan.

[5]Department of Hygienic Chemistry, Graduate School of Biomedical Sciences, Nagasaki University. 1-14 Bunkyo-machi, Nagasaki, 852-8521, Japan.

[6]Department of Radioisotope Medicine, Atomic Bomb Diseases Institute, Nagasaki University, 1-12-4 Sakamoto, Nagasaki, 852-8523, Japan.

[7]Mahara Institute of Medical Acarology, 56-3 korekuni Aratano-cho, Anan, Tokushima 779–1510, Japan.

*Corresponding author: Daisuke Hayasaka, Department of Virology, Institute of Tropical Medicine, Nagasaki University, 1-12-4 Sakamoto, Nagasaki, 852-8523, Japan. Phone number: +81-95-819-7828. Fax number: +81-95-819-7830. E-mail address: hayasaka@nagasaki-u.ac.jp

Supplementary figure 1

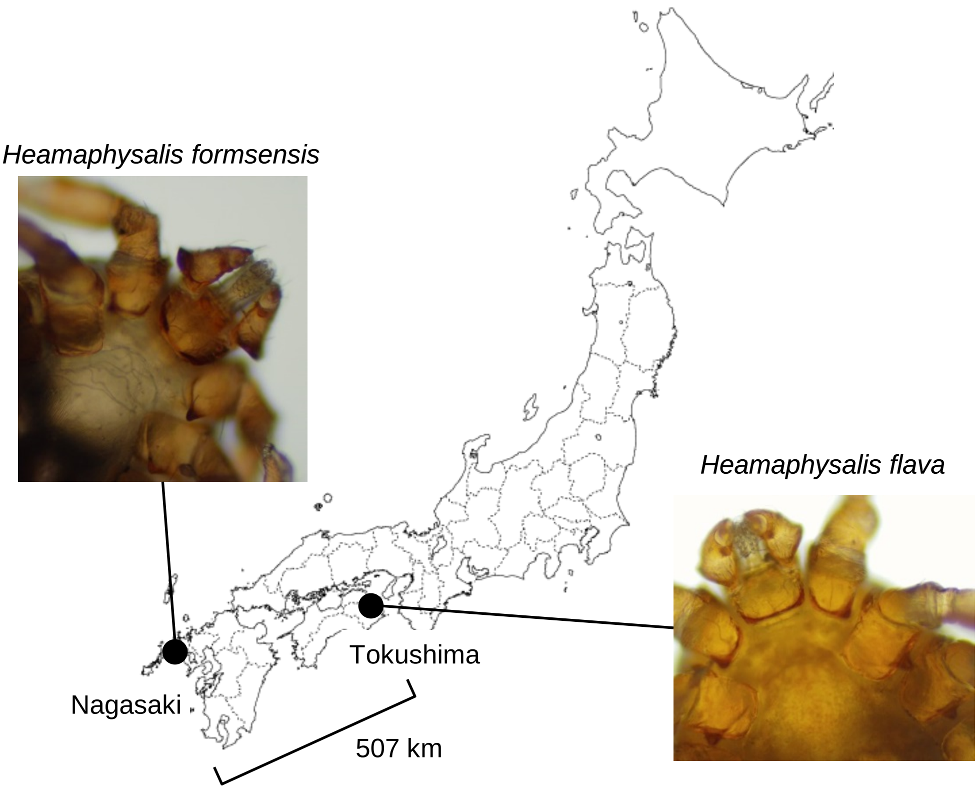

Supplementary figure 2

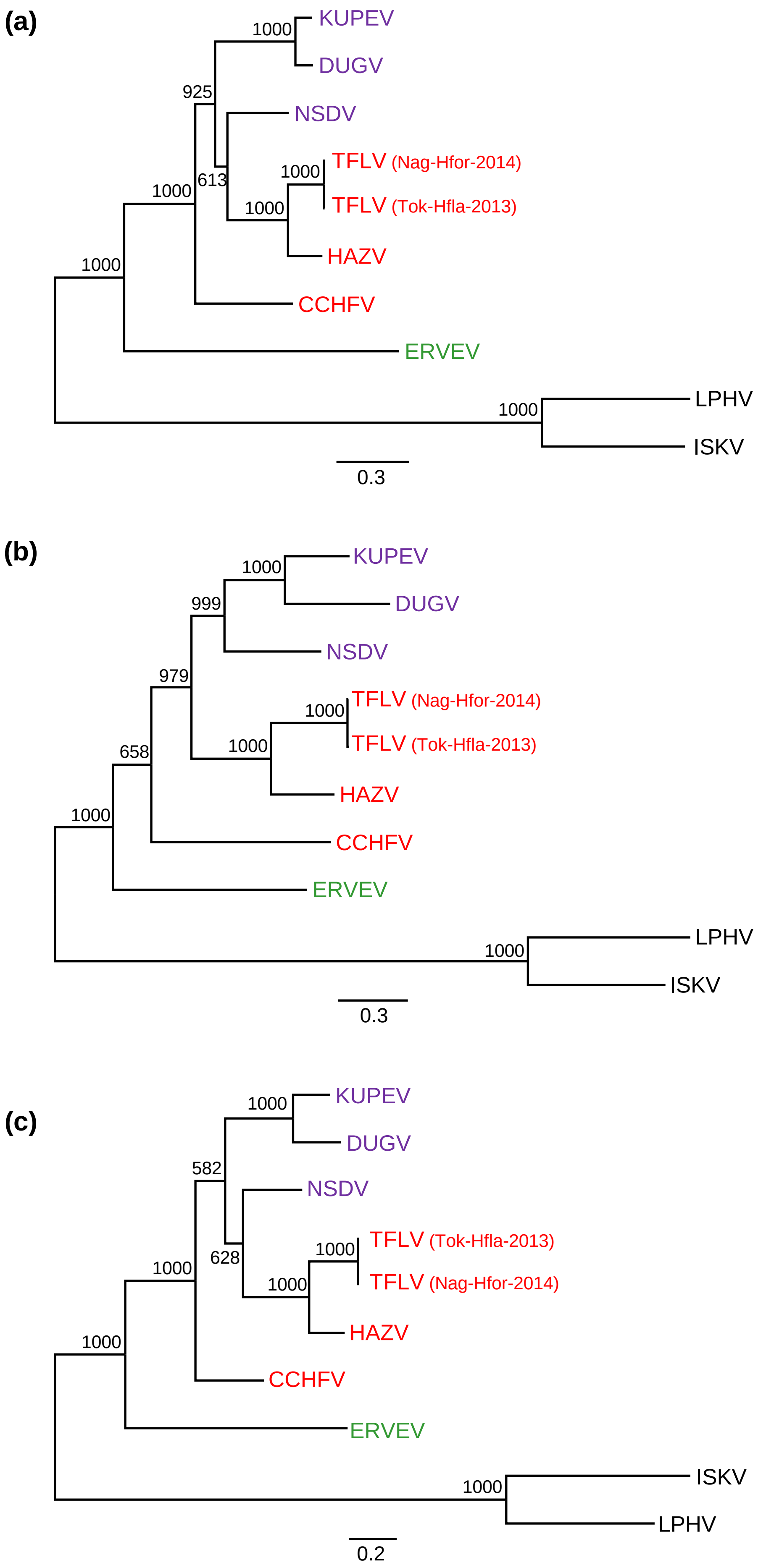

Supplement: Supplementary Figures [file srep20213-s1.pdf]
